# A Study on the Prediction of Operating Conditions in Mining Drilling Systems Using a Deep Temporal Forecasting Network

**DOI:** 10.3390/s26216753

**Published:** 2026-10-25

**Authors:** Shuang Xia, Renbo Yu, Guorui Liang, Lina Zhao, Yuanhang Qiu, Zheng Zhao, Kunhao Shi, Lijia Luo

**Affiliations:** 1 School of Information and Control Engineering, China University of Mining and Technology, Xuzhou 221116, China; x_shuang@cumt.edu.cn; 2 School of Mining, China University of Mining and Technology, Xuzhou 221116, China; tb24020040a41ld@cumt.edu.cn (R.Y.); lgr88285033@gmail.com (G.L.); ts25020254p31@cumt.edu.cn (L.Z.); 3 School of Computer Science and Technology/School of Artificial Intelligence, China University of Mining and Technology, Xuzhou 221116, China; 4 State Grid Jiangsu Electric Power Co., Ltd. Xuzhou Power Supply Branch, Xuzhou 221005, China; 361773646@163.com; 5 School of Mechanical and Electrical Engineering, China University of Mining and Technology, Xuzhou 221116, China; shikunhao@cumt.edu.cn (K.S.); 15695256587@163.com (L.L.)

**Keywords:** deep temporal forecasting network, drilling systems, operational status prediction, multiscale causal convolutional networks, attention mechanisms

## Abstract

Accurately predicting the operational status of mining drilling systems under complex coal and rock conditions remains challenging due to the intricate dynamic characteristics of drilling tools, highly variable operating conditions, and the pronounced long-term dependence associated with drill-jamming risk. To address these challenges, this study proposes a deep temporal prediction network for forecasting the operational status of mining drilling systems. Specifically, a multiscale dilated causal convolutional network is employed to extract temporal features at multiple scales, thereby capturing dependencies over different time horizons. Subsequently, a temporal convolutional network (TCN) integrated with an attention mechanism is used to model multidimensional temporal correlations among the extracted features and adaptively assign channel-wise weights, thereby emphasizing informative features while suppressing irrelevant information. This architecture enables the accurate prediction of the drill-jamming factor. Furthermore, the joint objective supplements pointwise prediction error with a global temporal-correlation preference. The experimental results demonstrate that the proposed method achieves satisfactory predictive performance and captures temporal variation within the evaluated drilling sequence.

## 1. Introduction

Coal remains a fundamental conventional energy resource and a strategic pillar of industrial activity and energy security in many economies [1]. The resource endowment of China is characterized by abundant coal but limited oil and natural gas, which gives coal a dominant role in the national energy security system [2,3]. In 2025, the raw coal output of China reached 4.85 billion tonnes, an increase of 1.4% over the previous year [4]. Against this background, mining drilling equipment has become an important means of improving extraction efficiency, controlling safety risks, and using resources more intensively in underground operations. The real-time monitoring and early warning of drill-string operating conditions are therefore essential for safe rig operation and efficient drilling [5]. The operating state of a coal mine drilling system has the following characteristics:

(1) Complex and variable coal seam conditions cause frequent transitions between operating regimes. The drilling parameters therefore exhibit pronounced multiscale behavior, and their dynamic characteristics change across temporal scales;
(2) As drilling depth increases, cuttings continue to accumulate, the cuttings removal pressure acting on the drill pipe rises, and the drill string is subjected to increasingly strong external disturbances;
(3) The combined effects of these factors produce strong long-term dependence in the risk of drill-string sticking, so operating state prediction must account for both temporal correlation and the underlying evolution trend.

Broader prognostic studies offer models but limited evidence for drilling. Liao’s CNN [6] did not test drill strings. Chen et al.’s graph model [7] used only two non-drilling datasets (a simulated turbine and a hydraulic test bench), leaving field transfer unknown. Wang et al.’s BiTCN-Informer [8] evaluated remaining useful life (RUL) on three aero-engine datasets, not sticking-factor forecasts; Lyu et al.’s transfer model [9] used one turbofan dataset, leaving transfer to drilling rigs untested. Zhao et al.’s DEM-MFBD model [10] simulated one MG325 shearer cutting a 17-layer coal wall at fixed settings, not time-varying drilling. Wang et al.’s particle-filter/EM method [11] used one experimental bearing run-to-failure case and reported slow EM convergence. Liu et al.’s time-lag DBN [12] used one CTCS-3 train control case and requires estimated lag relationships. Yin et al.’s BiLSTM-SOM-PSO [13] was tested on one bearing dataset; Elasha et al.’s ANN regression [14] was tested on 50 days of data from one turbine bearing, with RUL estimates oscillating near failure. Zhang’s PSO-BP model [15] concerns one shearer planetary carrier, without drill-string validation.

Drilling-specific work addresses sensing, detection, prediction, and warning, but each method leaves a gap. Liu et al. [5] monitored drill-string vibration online without reporting a future sticking forecast. Zou et al. [16] used hierarchical cointegration to monitor sticking while drilling four uniform test stones; detection results do not establish advance warning time. Wu et al. [17] predicted a sticking factor using data from one drilling simulation platform and set a critical line, but reported neither underground-field error nor event-level lead time. Chen et al. [18] trained WOA-KELM with 370 samples from one borehole; despite the 100% test recognition of sticking conditions, this classifies observed states rather than forecasting future events. Dai et al. [19] obtained R^2^ = 0.963 and MSE = 29.742 for drilling-rate prediction, but did not predict sticking risk or its warning horizon. Their later drillability-based controller [20] reported 84% state-identification accuracy and 31.9% rotational-speed overshoot, without evaluating prospective sticking forecasts. Zhang et al. [21] demonstrated multisource sensing and sticking-condition prediction at two mines, including three lithology classes in one field application, but did not report horizon-specific sticking factor error or event-level warning lead time. Together, these studies leave sticking factor prediction over specified horizons and actionable warning lead time insufficiently validated.

These limitations motivate a model that captures multiscale variation and long-range dependence in the sticking-factor series. The present study evaluates forecasting accuracy on an experimental sequence; event-level warning lead time and underground field performance require separate validation.

To address these challenges, this study develops a deep temporal forecasting network for predicting the operating state of a mining drill string. First, multiscale dilated causal convolution (MDCC) is used to extract multiscale features generated by multiple operating regimes under complex coal rock conditions. Second, a temporal convolutional network (TCN) models within-scale temporal dependence and strengthens the representation of long-range relationships. Third, squeeze-and-excitation attention (SE attention) adaptively reweights feature channels according to their importance across scales, dynamically enhancing informative features and improving operating state identification and prediction. In addition, a temporal trend constraint is incorporated into the loss function to introduce a global temporal preference alongside pointwise error minimization. The proposed method is finally evaluated experimentally and compared with established alternatives.

## 2. Architecture of the Deep Temporal Forecasting Network

### 2.1. Overall Architecture

**Figure 1:**
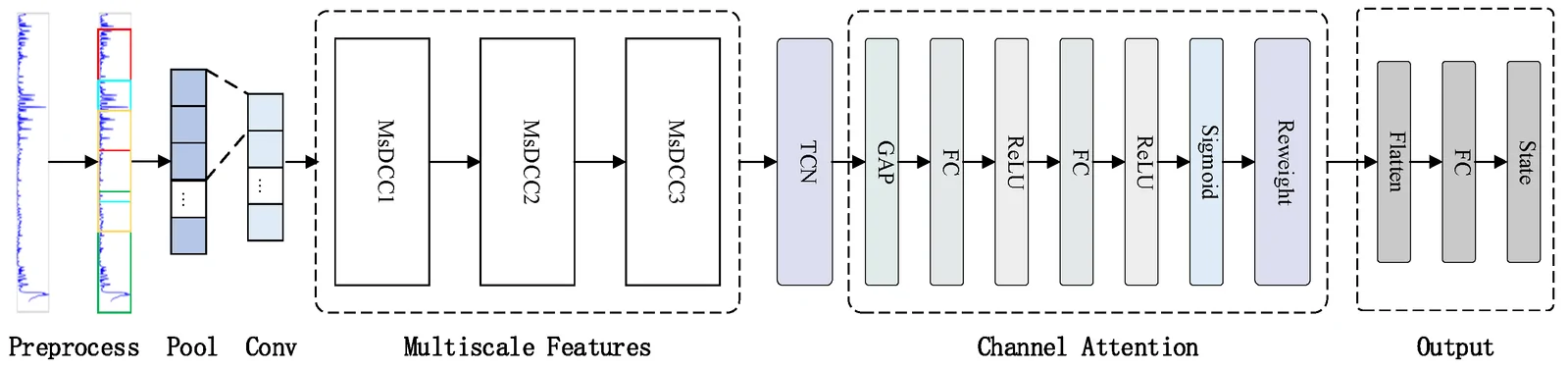
Overall structure of drilling tool sticking state prediction.

During the operation of a mining drilling rig, the complex coal rock environment produces multiple operating regimes and markedly different dynamic responses, making operating state prediction difficult. Temporal modeling is complicated because drilling depth, changes in formation structure, drill-string wear, and other factors affect the system over extended periods. The proposed operating state prediction framework is shown in Figure 1. To capture the multiscale behavior caused by transitions among operating regimes, the framework first applies MDCC to extract features and dependencies at different temporal scales. As drilling depth increases, the disturbance range of the drill pipe expands and the system exhibits complex multidimensional temporal correlations. A TCN is therefore used to strengthen temporal modeling and improve prediction accuracy. SE attention then adaptively adjusts the weight of each channel, emphasizing informative features and suppressing irrelevant noise. These components jointly enable the accurate prediction of the drilling system operating state.

The detailed model configuration is listed in Table 1.

The input data first pass through a max-pooling layer with a 1 × 2 kernel and a stride of 3, and then through a causal convolution layer with a 1 × 64 kernel. The large convolution kernel captures a broad range of signal features. The resulting feature sequence then enters three cascaded multiscale dilated causal convolution networks. Each network applies ReLU activation and batch normalization (BN), followed by three parallel DCC branches with dilation factors d = 1, 2, and 3 to extract features at different scales. This max-pooling layer reduces computation before the MDCC blocks. The residual path uses a standard 1 × 1 convolution with a stride of 3. Because the pooling stride exceeds its kernel width, not every intermediate feature position is directly covered. Brief pre-sticking precursors may therefore be lost; the present experiments assess forecasting accuracy rather than event-level precursor recall and cannot establish the complete preservation of such events. A controlled comparison with smaller pooling strides (1 or 2) or overlapping pooling, using event-level precursor recall as an evaluation criterion, is therefore needed before the stride-3 configuration can be considered suitable for early-warning applications. After the three cascaded MDCC networks have extracted multiscale features, the data are passed to a TCN with a 1 × 3 kernel and 64 filters to capture long-range dependencies. SE attention is incorporated into the TCN to dynamically reweight feature channels and further enhance informative features. Global average pooling and a fully connected layer then produce the operating-state prediction.

To improve prediction of the operating state of the mining drilling system and introduce a global temporal preference, a temporal trend constraint is added to the conventional mean squared error loss to form the following joint objective:

(1) $$L=\lambda_{1}L_{pred}+\lambda_{2}L_{trend}$$

Here, $L_{pred}$ is the mean squared prediction error loss defined in Equation (2). The coefficients λ_1_ and λ_2_ weight the prediction error and trend terms, respectively. The sign and relative magnitude of λ_2_ determine the direction and strength of the correlation preference. Suitable values depend on validation performance. In the reported experiments, λ_1_ = 1 and λ_2_ = −0.1 were used, so minimizing the joint loss favors positive correlation between the predicted sticking factor and time, reflecting the overall progression toward sticking.

(2) $$L_{pred}=\frac{1}{M}\sum_{i=1}^{M}{\left(y_{i}-{\hat{y}}_{i}\right)}^{2}$$

In Equation (2), $y_{i}$ is the observed value, ${\hat{y}}_{i}$ is the corresponding prediction, and M is the sequence length; the subscript *i* indexes its samples.

A trend-correlation term is introduced to improve the model’s ability to represent time-series evolution. Equation (3) defines this term as the Pearson correlation between the predicted values and their time indices. This global signed measure does not directly penalize variation between adjacent predictions, so it alone cannot guarantee smoother forecasts.

(3) $$L_{trend}=\frac{\sum_{i=1}^{M}\left({\hat{y}}_{i}-\bar{\hat{y}})(i-\bar{i}\right)}{\sqrt{\sum_{i=1}^{M}{\left({\hat{y}}_{i}-\bar{\hat{y}}\right)}^{2}\sum_{i=1}^{M}{\left(i-\bar{i}\right)}^{2}}}$$

Here, $\bar{\hat{y}}$ is the mean of the *M* predicted values, and $\bar{i}$ is the mean of the corresponding time indices.

### 2.2. Multiscale Feature-Extraction Module

The coal-rock environment encountered by a mining drilling rig is complex and continuously changing, so the drill string exhibits dynamic behavior under multiple operating regimes. Corresponding time-series features vary across temporal scales. Multiscale feature extraction is therefore essential for accurately characterizing drill-string operation under these conditions. By extracting and fusing information at multiple scales, a model can represent both short-term fluctuations and long-term trends, improving its ability to describe complex patterns. This capability is particularly important for long sequences, for which single-scale models often fail to capture hierarchical structure and consequently provide limited accuracy and generalization.

#### 2.2.1. Temporal Convolutional Network

During drilling, disturbances acting on the drill string intensify as depth increases, making the time-series signals that describe its operating state increasingly complex. The temporal convolutional network (TCN) [22,23] is well suited to such data. Causal and dilated convolutions let the TCN capture long-range dependencies without using future inputs.

(1) Causal convolution

**Figure 2:**
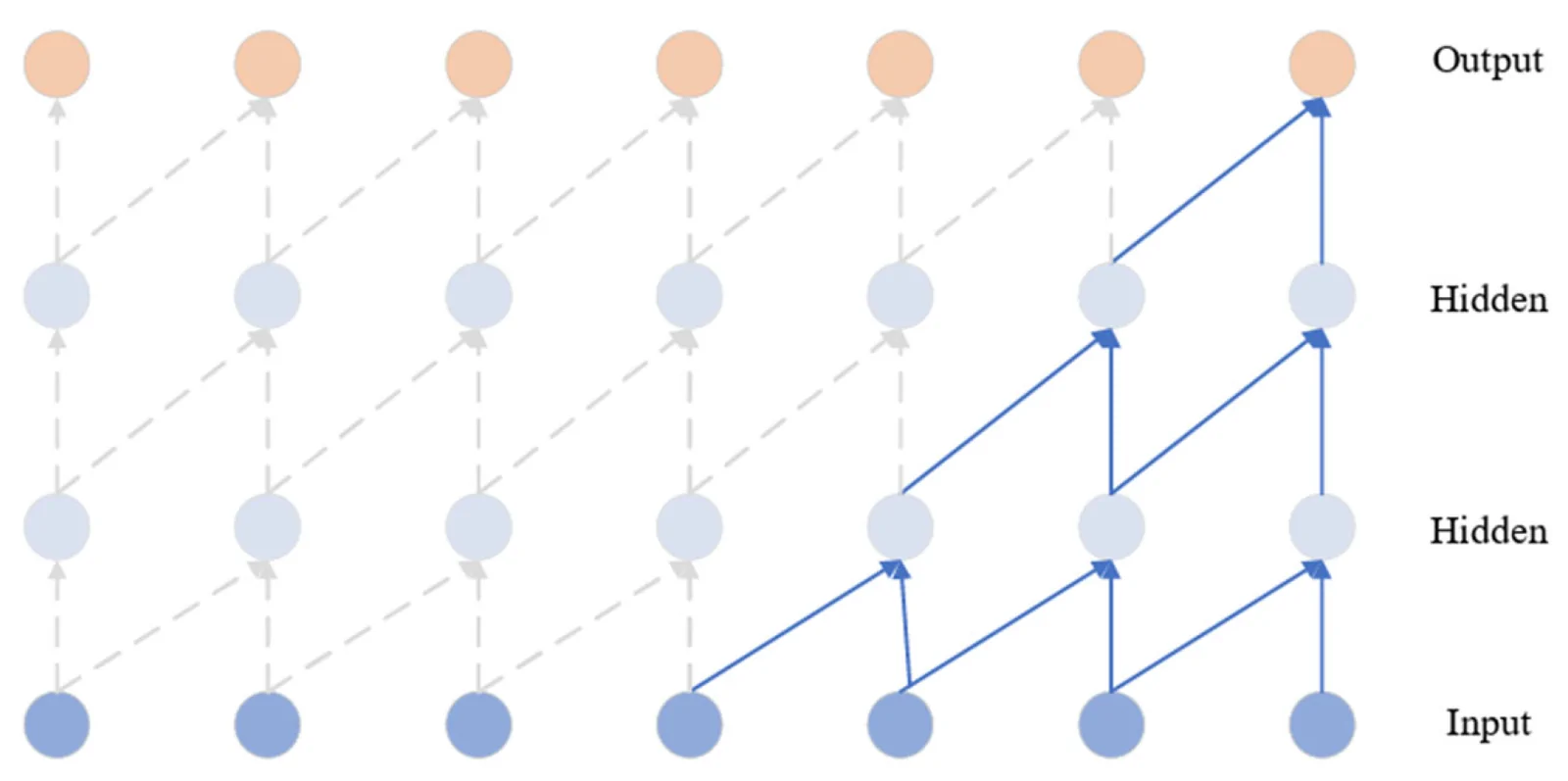
Schematic diagram of causal convolution network.

For time-series forecasting, current predictions must not use future inputs. Causal convolution, a core component of the TCN, restricts the convolution window and uses zero padding so that each output depends only on the current and previous inputs, preventing the leakage of future information. The operation is illustrated in Figure 2.

A limitation of causal convolution is its relatively small receptive field, which becomes increasingly restrictive as the sequence length grows. Dilated convolution is therefore introduced into the TCN to expand the receptive field and capture longer-range dependencies.

(2) Dilated convolution

**Figure 3:**
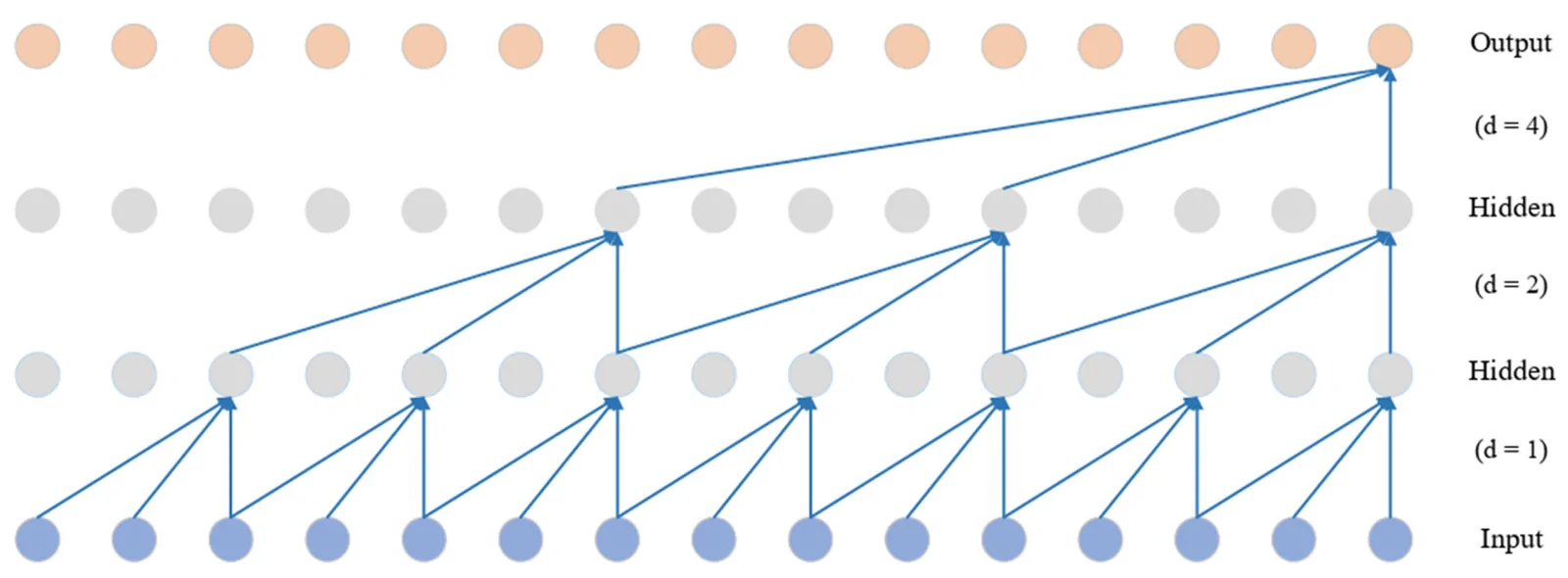
Schematic diagram of dilated convolutional network.

Dilated convolution, also known as atrous convolution, inserts gaps between kernel elements and thereby expands the receptive field without increasing the number of parameters. The dilation factor d controls the spacing between kernel elements and hence the receptive field size. As depth increases, exponentially increasing dilation factors allow the receptive field to grow rapidly without requiring many additional layers. The model can thus process information over a long temporal span with relatively few layers, as illustrated in Figure 3.

Unlike pooling, dilation skips positions within each kernel but retains all input samples for neighboring operations.

(3) Residual connections

As a deep neural network becomes deeper, training is increasingly susceptible to vanishing or exploding gradients. During backpropagation through many layers, gradients may shrink rapidly or grow without bound, preventing convergence and degrading training performance. The problem is especially pronounced in deep networks because the gradient at each layer depends on values propagated through all subsequent layers.

**Figure 4:**
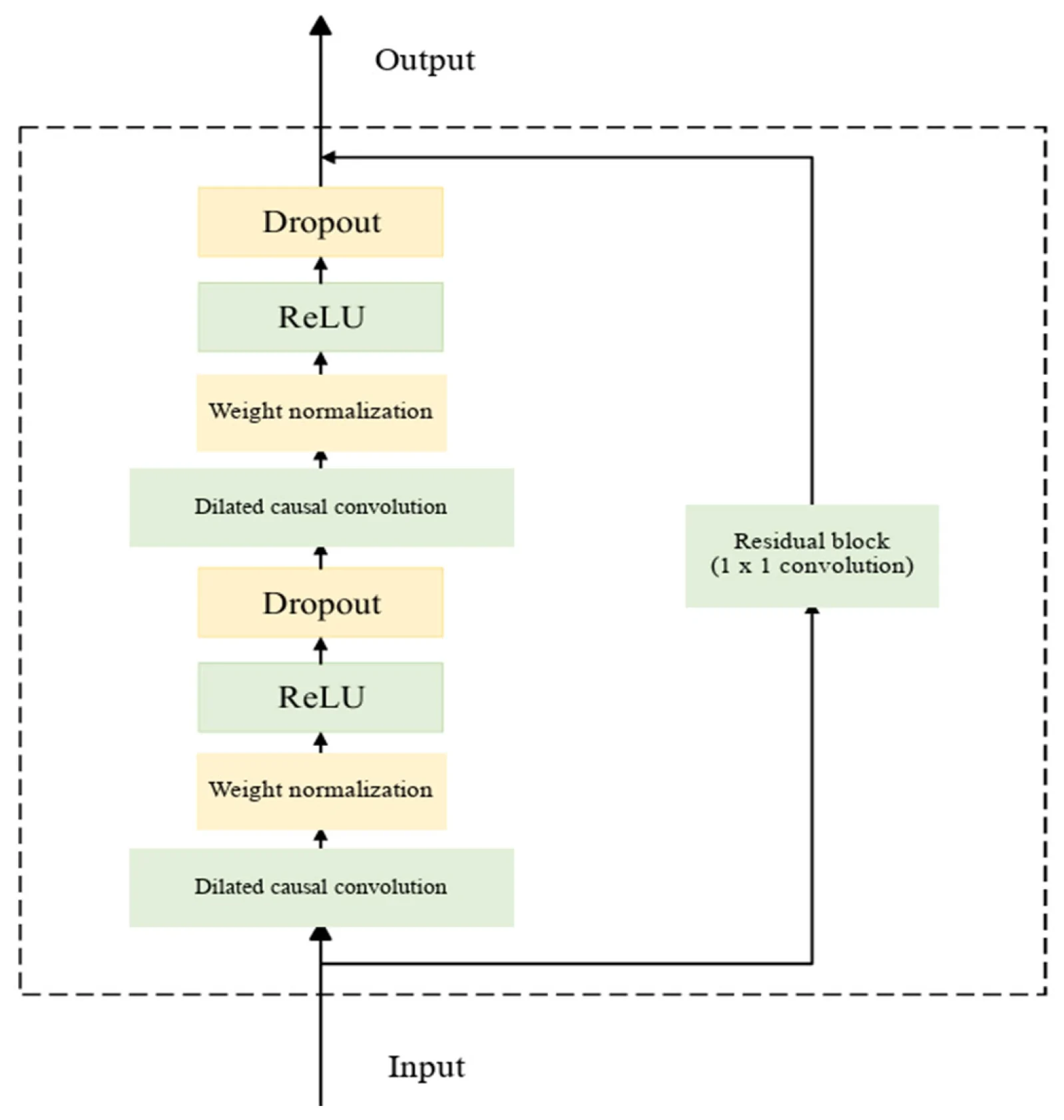
Schematic diagram of TCN.

TCNs use residual, or skip, connections to address this problem. A residual connection passes information directly to deeper layers instead of relying exclusively on sequential propagation. This design preserves the original signal in deep networks and makes identity mappings easier to learn. The TCN and its residual block are illustrated in Figure 4.

In a TCN residual block, the input x passes through two dilated-convolution layers, normalization, and activation to produce an intermediate feature map F(x). The original input x is then added to this feature map to yield the final output y = F(x) + x. The skip path allows information to pass directly from shallow to deep layers and reduces information loss. If the input and output dimensions differ, a linear transformation is applied to x so that the dimensions are compatible for elementwise addition.

#### 2.2.2. Multiscale Dilated Causal Convolution

Because the coal-rock environment is complex and variable, drilling proceeds through multiple operating regimes, producing signal features that change across scales. Multiscale feature extraction is therefore necessary for the accurate prediction of drill-string operating conditions. Dilated causal convolution can capture long-range dependencies within the TCN framework, but a single dilation scale may be insufficient for complex applications. It can limit the extraction of hierarchical features, weaken the representation of complex time-series dynamics, and perform poorly when the relevant phenomena occur at different temporal scales.

**Figure 5:**
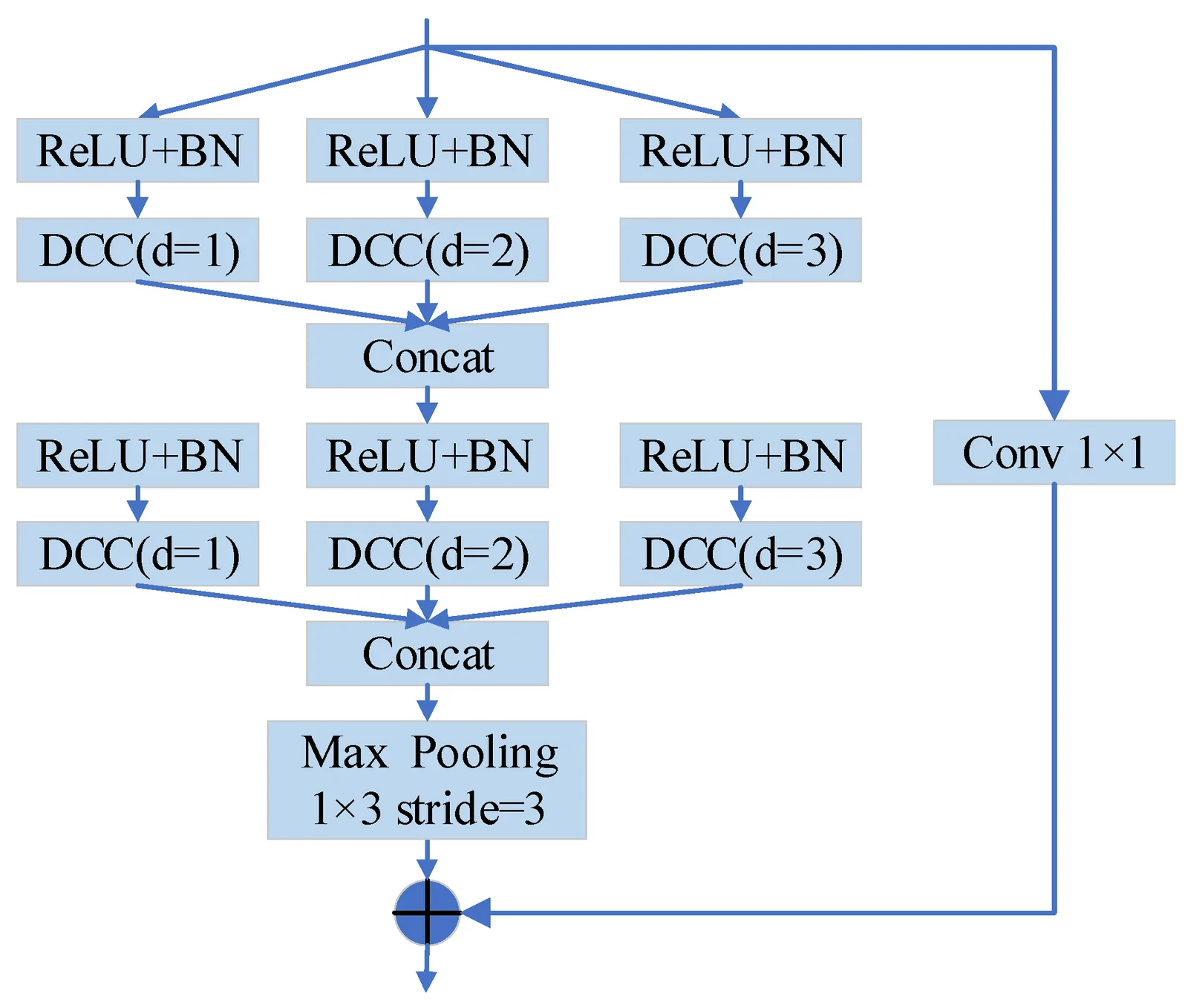
Schematic diagram of multiscale dilated causal convolution.

To overcome this limitation, an enhanced strategy known as multiscale dilated causal convolution has been proposed [24]. It combines convolution branches with different dilation factors so that local fine-grained information and global long-range dependencies can be captured simultaneously. By processing a time series with different receptive fields, the model extracts richer features across temporal scales, as illustrated in Figure 5.

As shown in Figure 5, multiscale dilated convolution extends the TCN with several parallel dilated branches. Different dilation factors give the kernels different temporal spans: small factors capture short-term local features, whereas large factors capture long-term global dependencies. The parallel feature maps are fused in a subsequent layer, strengthening the multiscale representation. This architecture improves the modeling of both short- and long-range dependence without a substantial increase in computational complexity, thereby supporting more accurate operating state prediction.

### 2.3. Channel-Attention Module

Because mining drilling signals respond at multiple scales as operating conditions change, the multiscale features extracted by the preceding module must be weighted appropriately to predict the evolution of the drill-string operating state. Deep models are now widely used for time-series forecasting, but they must emphasize components that materially affect the prediction while suppressing irrelevant noise. Attention mechanisms address this requirement by assigning data-dependent weights to different portions of the input, allowing a model to focus on informative content. For time-series data, attention can reveal relationships among time steps and improve prediction accuracy and reliability.

**Figure 6:**
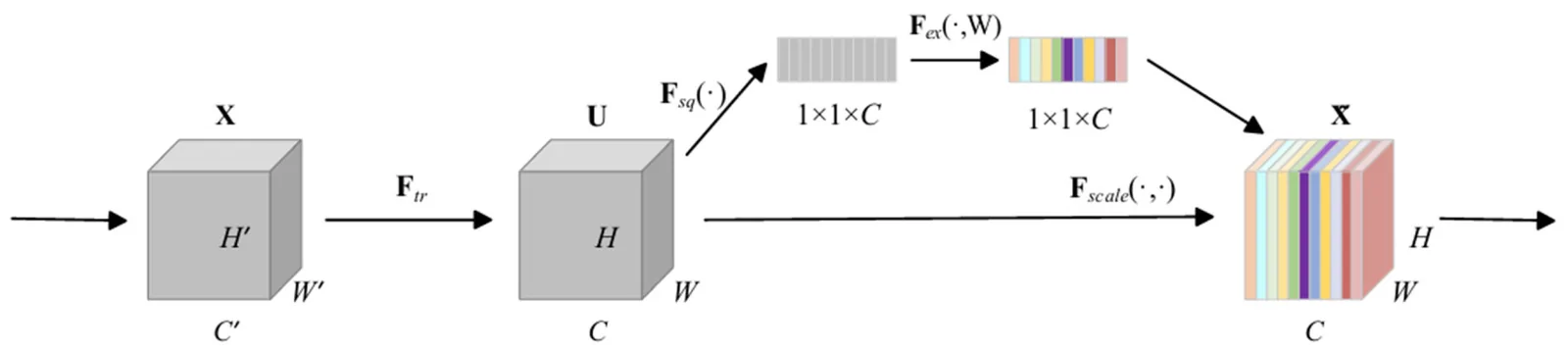
Schematic diagram of SE attention mechanism.

Squeeze-and-excitation (SE) attention [25] is an effective mechanism for strengthening feature representations. It adaptively models interchannel relationships and dynamically reweights feature channels, emphasizing those that contribute to the task while suppressing irrelevant or redundant information. SE attention comprises squeeze and excitation stages. The squeeze stage applies global average pooling to each channel of the input feature map, aggregating spatial information into a global channel descriptor. The excitation stage then uses fully connected layers and activation functions to learn a weight for each channel. These weights are applied to the input feature map to enhance informative channel responses. The network structure is shown in Figure 6.

Let the input feature be $X\in R^{C^{\prime }\times T^{\prime }}$, where $C^{\prime }$ and $T^{\prime }$ denote the input channel count and sequence length, respectively. A one-dimensional temporal convolution transforms the input into $U\in R^{C\times T}$, where C and T denote the output channel count and sequence length. The mapping is defined as follows:

(4) $$U_{c}=V_{c}*X=\sum_{k=1}^{C^{\prime }}V_{c}^{k}*X^{k}$$

In Equation (4), $V_{c}$ contains the parameters of the c-th output filter, and $V_{c}^{k}$ is the one-dimensional temporal kernel for input channel k. Here, ∗ denotes convolution along time; $X^{k}$ and $U_{c}$ are the corresponding input and output channel sequences.

Global average pooling then compresses the temporal dimension, producing a length-C vector $z\in R^{C}$. Each element $z_{c}$ represents the mean response of channel c over all T time steps. This operation is expressed as:

(5) $$z_{c}=F_{\mathrm{sq}}(U_{c})=\frac{1}{T}\sum_{t=1}^{T}U(c,t)$$

This operation reduces each channel to a single scalar descriptor. The time index t spans 1,…,T, and c indexes the C output channels.

In the excitation stage, the global descriptor z is used to learn the importance of each channel. Two fully connected layers with an intervening activation function are typically employed. The first layer reduces the original C channels to C/r channels, lowering computational cost and helping to prevent overfitting. A ReLU activation introduces nonlinearity. The second layer restores the channel dimension to C, and sigmoid mapping produces the channel weight vector $s\in R^{C}$:

(6) $$s=F_{\mathrm{ex}}(z,W)=\sigma (W_{2}\delta (W_{1}z))$$

Here, $W_{1}\in R^{\left(C/r\right)\times C}$ and $W_{2}\in R^{C\times \left(C/r\right)}$ are the weight matrices of the two fully connected layers. The reduction ratio r controls the intermediate channel count; δ denotes ReLU and σ denotes the sigmoid function.

Finally, the learned channel weights are applied to the corresponding features to produce the output $\tilde{X}\in R^{C\times T}$:

(7) $${\tilde{x}}_{c}(t)=F_{\mathrm{scale}}(u_{c}(t),s_{c})=s_{c}\times u_{c}(t)$$

In Equation (7), $u_{c}\left(t\right)=U(c,t)$ is the value of channel c at time step t, and $s_{c}$ is its learned scalar weight. The same weight is applied across all T time steps of that channel.

## 3. Experimental Validation and Results

Experiments are conducted to assess the effectiveness and reliability of the proposed method for prediction of mining drill-string operating conditions. Performance is evaluated on an instrumented drilling test platform simulating coal rock drilling and compared with related methods. The experiments examine both the ability to describe the current operating state and the accuracy of future-state prediction, thereby quantifying improvements in forecast accuracy and robustness.

### 3.1. Evaluation Metrics

Model performance is evaluated quantitatively using the following regression metrics.

Coefficient of determination R^2^: This metric measures goodness of fit. A value closer to 1 indicates that the model explains more of the observed variance and fits the data more closely.

(8) $$R^{2}=1-\frac{\sum_{i=1}^{N}{\left({\hat{x}}_{i}^{c}-x_{i}^{c}\right)}^{2}}{\sum_{i=1}^{N}{\left({\bar{x}}_{i}^{c}-x_{i}^{c}\right)}^{2}}$$

Mean absolute error (MAE): the mean absolute difference between predicted and true values, which quantifies the average error magnitude.

(9) $$MAE=\frac{1}{N}\sum_{i=1}^{N}\left|{\hat{x}}_{i}^{c}-x_{i}^{c}\right|$$

Mean squared error (MSE): the mean squared difference between predicted and true values.

(10) $$MSE=\frac{1}{N}\sum_{i=1}^{N}{\left({\hat{x}}_{i}^{c}-x_{i}^{c}\right)}^{2}$$

Mean absolute percentage error (MAPE): the mean prediction error expressed as a percentage of the true value.

(11) $$MAPE=\frac{1}{N}\sum_{i=1}^{N}\frac{\left|{\hat{x}}_{i}^{c}-x_{i}^{c}\right|}{x_{i}^{c}}\times 100\%$$

Here, ${\hat{x}}_{i}^{c}$ is the predicted value at the i-th sample, and $x_{i}^{c}$ is the corresponding true value at the i-th sample. N denotes the number of samples used to calculate each metric.

Root mean squared error (RMSE): the square root of the mean squared error, which represents the average dispersion of predictions from the true values.

(12) $$RMSE=\sqrt{\frac{1}{N}\sum_{i=1}^{N}{\left({\hat{x}}_{i}^{c}-x_{i}^{c}\right)}^{2}}$$

### 3.2. Experimental Results

The dataset comprises multiple drilling records. The illustrated test record was acquired at 10 kHz, corresponding to a raw acquisition interval of 0.1 ms, over approximately 57.7 s. During preprocessing for constructing the monitoring statistic used in prediction, each series of this record was reduced to 5770 points. The raw acquisition interval should be distinguished from the interval between samples supplied to the prediction model.

The instrumented test platform used four sensors: a pressure sensor, a torque sensor, a laser displacement sensor, and a triaxial accelerometer. They recorded feed pressure, torque, drilling depth, and vibration in three directions. In this record, each of the four measured data types comprised 577,002 raw time points. The record contains an extended normal drilling interval, with the sticking event occurring at approximately 56.1 s. The prediction target, termed the sticking factor, is the scalar global T^2^ monitoring statistic obtained through hierarchical fusion of sensor-derived drilling features, following Zou et al. [16].

With L = 100 processed input samples at Δt = 0.01 s (100 Hz), the model predicts the next K = 100 consecutive samples. This gives a horizon H = KΔt = 1 s and a first-to-last input span of (L − 1)Δt = 0.99 s. Table 1 lists these settings.

**Figure 7:**
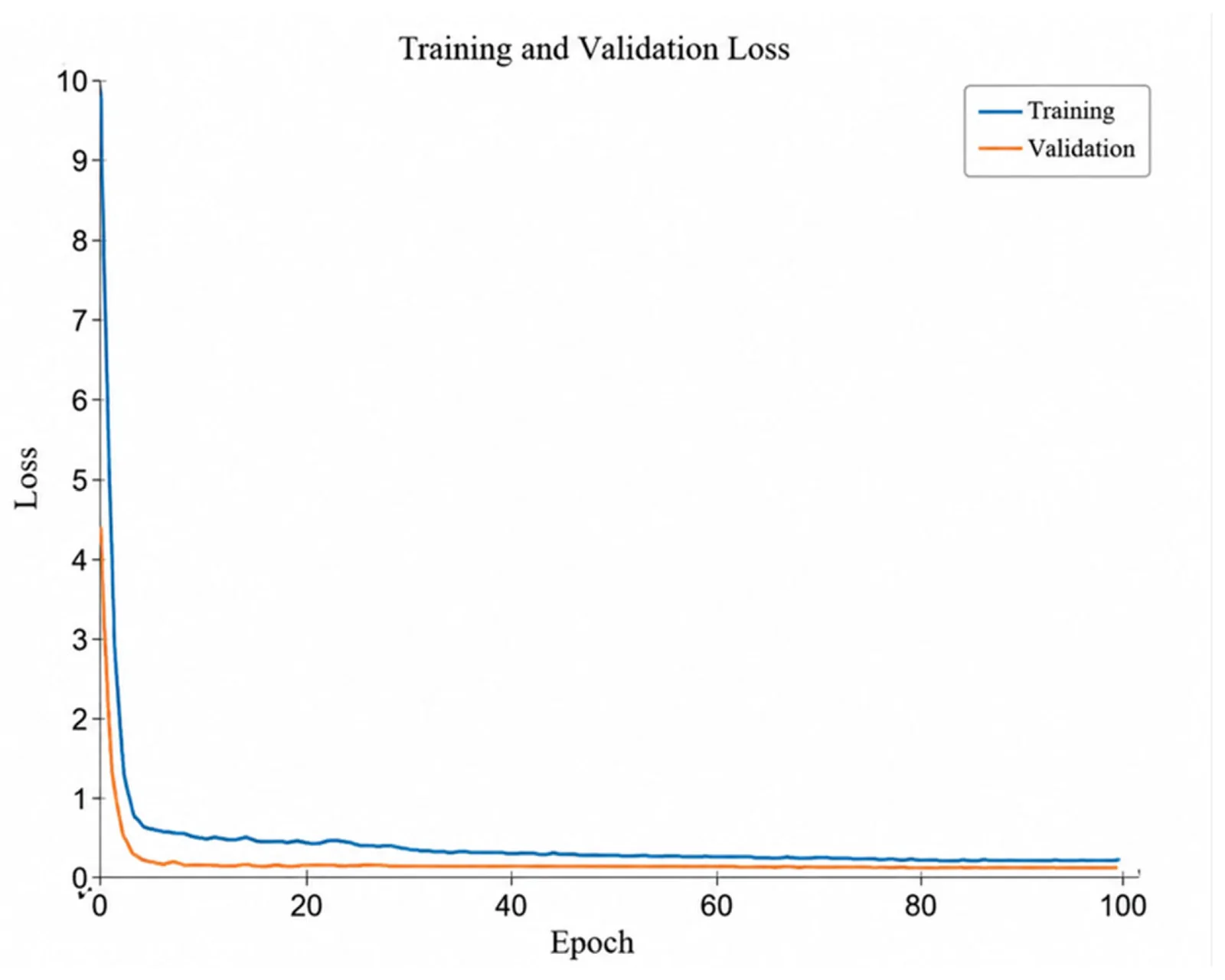
Training and validation loss.

**Figure 8:**
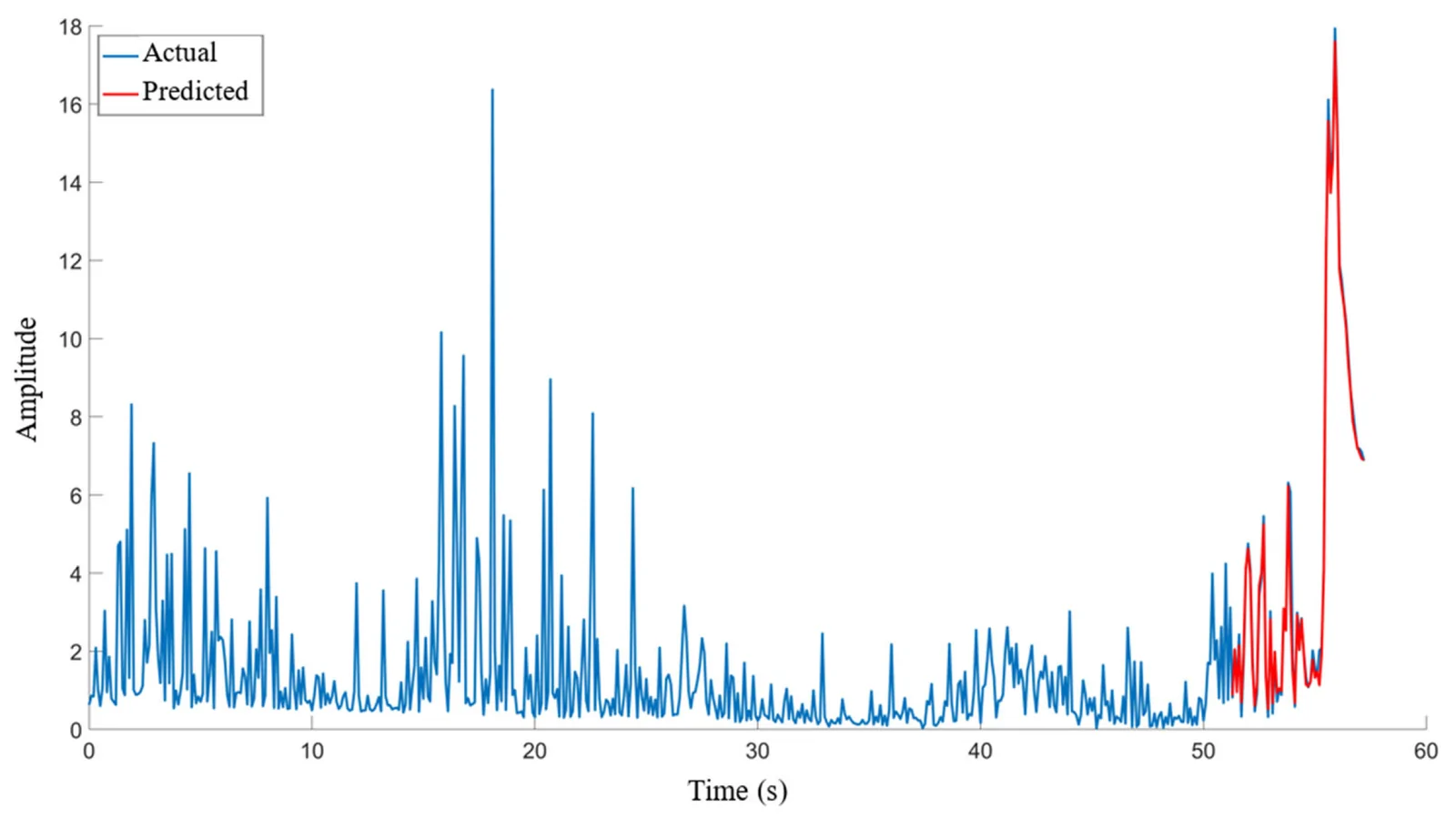
Prediction results of MsTCN-SE model.

Complete records are split chronologically into training, validation, and test sets (7:2:1), with windows confined to their assigned record and set. No additional sample gap is imposed; forecasting normalization parameters are fitted on training data only and applied unchanged to validation and test data. The Adam optimizer is used with a learning rate of 0.0005. A dropout rate of 0.3 is applied to reduce overfitting, and the model is trained for 100 epochs. The loss curves are shown in Figure 7. Loss decreases rapidly during the initial epochs, indicating that the model quickly captures the dominant patterns in the data. The rate of decrease then slows and the curves converge smoothly. The final loss is 0.1148, indicating a satisfactory fit to the training data. After training and optimization, the MsTCN-SE model is used to forecast the sticking factor; the results are shown in Figure 8.

Figure 8 shows a held-out test record on a 0–60 s axis. The red predictions span approximately the final 6 s of this record and follow the blue measured curve. Table 2 reports metrics calculated on test data only. This displayed span differs from the input look-back span, (L − 1)Δt for L input samples, and forecast lead, H = KΔt for K future steps, where Δt denotes the model sample interval.

### 3.3. Comparative Experiments

**Figure 9:**
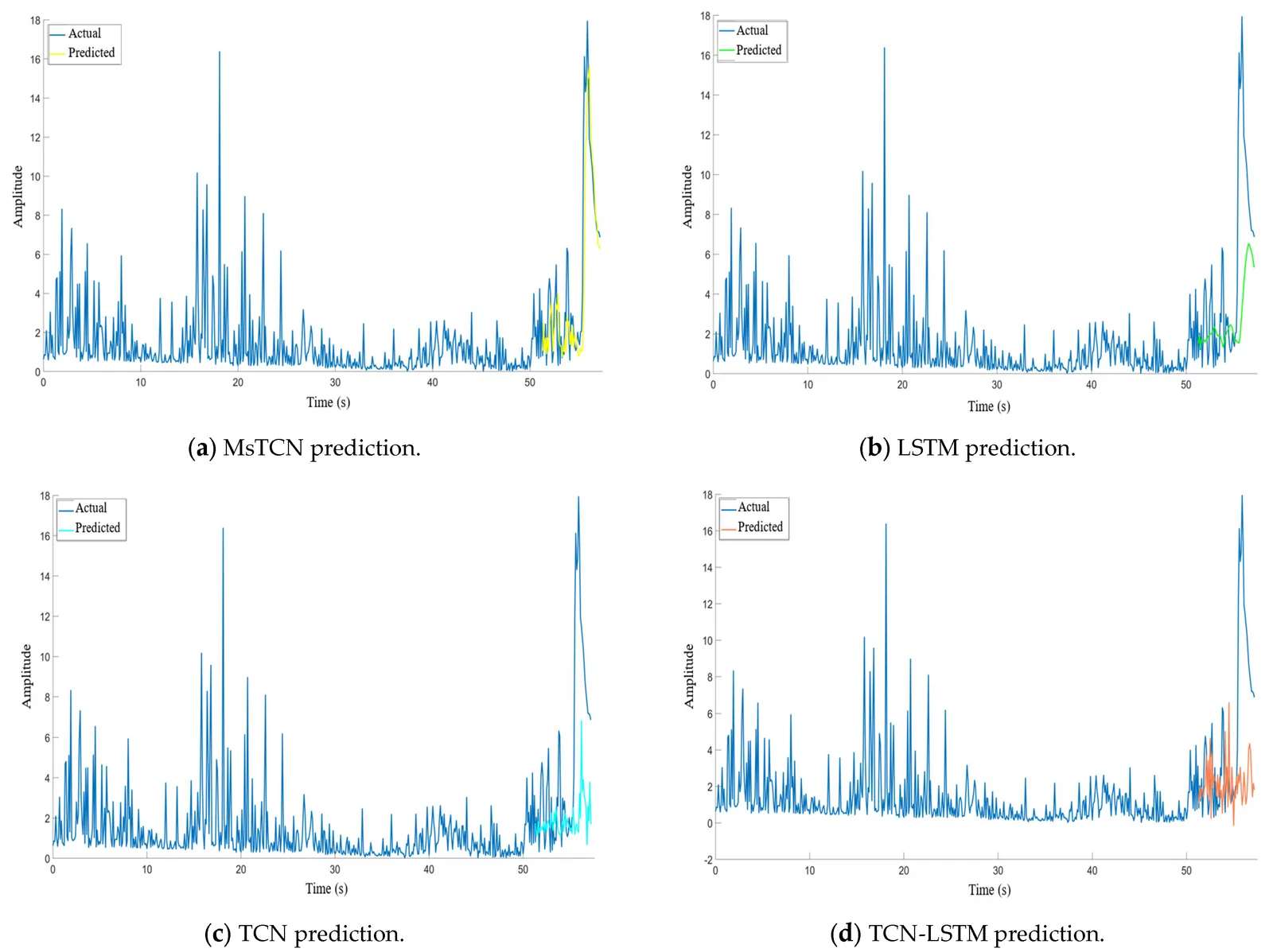
Comparative experiment prediction results.

Comparative experiments are performed to further evaluate the effectiveness and accuracy of the proposed model. The baselines are a multiscale temporal convolutional network (MsTCN), an LSTM, a TCN, and a hybrid TCN-LSTM. Their prediction results are shown in Figure 9.

Among the baseline models in Figure 9, MsTCN provides the best predictions. It captures both local variations and large excursions in the sticking factor, demonstrating strong temporal modeling capability. In contrast, TCN-LSTM performs poorly on this dataset and fails to reproduce either the local fluctuations or the large-scale changes, leading to substantial errors. This result suggests that, for these data, TCN-LSTM has a limited ability to represent both local and long-range dependencies.

The proposed method and the comparison models are evaluated quantitatively using the metrics reported in Table 2. The performance metrics reported in Table 2 are averages over multiple experimental runs.

The results are analyzed in terms of prediction accuracy and computational cost. Standard regression metrics and their normalized counterparts are used to compare the models, followed by an assessment of single-prediction latency.

Table 2 shows that MsTCN-SE performs best on all five metrics. Its coefficient of determination is the largest and is close to 1, while all four error metrics are the smallest. The negative R^2^ values of TCN and TCN-LSTM indicate that their sums of squared prediction errors exceed those of a constant predictor using the mean of the observed test values, but do not necessarily imply training non-convergence. These results demonstrate improved operating-state prediction accuracy for the mining drill-string on the evaluated dataset. The comparison between MsTCN and MsTCN-SE does not isolate the contributions of SE attention and the trend term, and controlled ablation experiments are needed to quantify their individual effects. The comparative advantage demonstrated in this study is limited to the selected baseline models and the evaluated dataset. Further benchmarking against a broader range of models is needed to determine whether this advantage extends to other forecasting methods.

For a more intuitive comparison, each metric is mapped to a normalized interval to obtain adjusted R-squared (AR^2^), adjusted MAE (AMAE), adjusted MSE (AMSE), adjusted MAPE (AMAPE), and adjusted RMSE (ARMSE). For every adjusted metric, a value closer to 1 indicates better predictive performance. The results are listed in Table 3.

**Figure 10:**
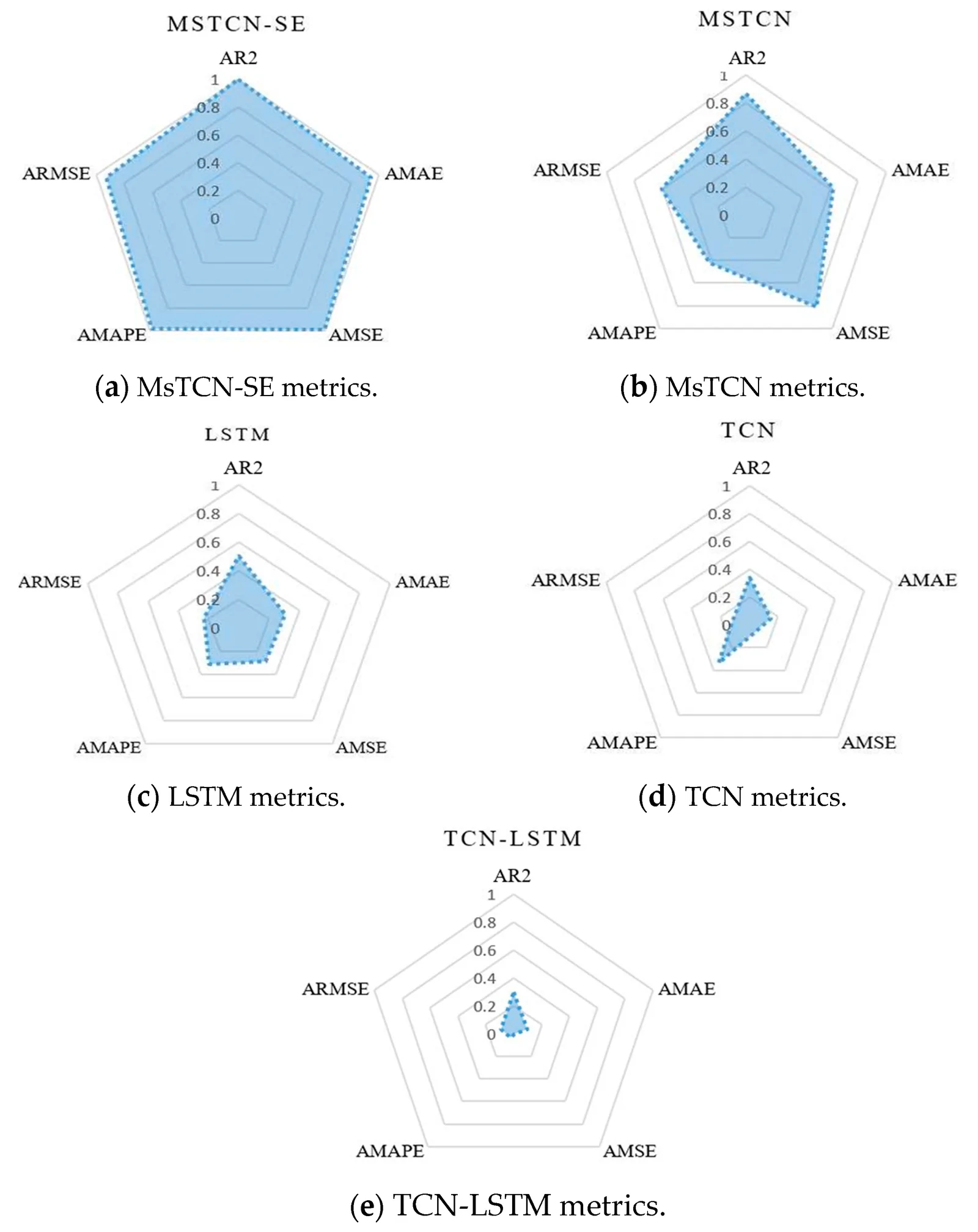
Radar chart of evaluation indicators for each prediction model.

The adjusted metrics are visualized using radar charts in Figure 10. The polygon corresponding to MsTCN-SE covers the largest area. Because a larger area represents stronger aggregate performance across the metrics, the visualization confirms that the proposed model substantially outperforms the alternatives.

MsTCN-SE requires 0.41 s per prediction (Table 4), leaving approximately 0.59 s before the 1 s horizon endpoint, excluding other system delays. This may permit fast automatic responses within that budget; manual intervention depends on operator and equipment response times. Inference spans 41 model sampling intervals, requiring an appropriate update schedule or faster inference. The demonstrated forecasting accuracy motivates subsequent system-level warning validation.

## 4. Conclusions

This study proposes an MsTCN-SE model for predicting the operating state of a mining drilling system under multiple operating regimes with complex multidimensional and long-range temporal correlations. The model first uses multiscale dilated causal convolution to extract scale-dependent signal features and represent dynamic responses under complex coal rock conditions. A TCN then strengthens long-range temporal modeling, while SE attention adaptively reweights feature channels to enhance informative state features and suppress redundant noise. A temporal trend constraint is also incorporated into a joint loss, adding a global temporal preference to pointwise error minimization. Experiments show that MsTCN-SE outperforms the comparison models on every evaluated metric. It achieves an R-squared value of 0.9915, and its MAE and RMSE are substantially lower than those of mainstream models such as LSTM and TCN. The experimental findings are limited to sticking factor forecasting on the evaluated drilling sequence and do not establish generalization across different rock samples, operating settings, or drilling equipment. Event-level precursor recall was not evaluated, so the effect of stride-3 max pooling on brief pre-sticking events remains unresolved and requires assessment with smaller strides or overlapping pooling.

The proposed method offers a promising approach to sticking factor forecasting, supported by the accuracy achieved on the evaluated drilling sequence. However, it is not yet a validated early-warning system. Alarm thresholds, event-level recall, warning lead time, and transferability to new mine conditions have not been verified in this study. Dedicated event-level and field validation is required before the method can be used for operational early warning. The dataset contains only one sticking event, and event-level warning lead time has not been quantified. Statistical assessment across events requires additional drilling records containing multiple sticking events.

## Figures and Tables

**Table 1:** Parameter configuration of MsTCN-SE.

Layer	Kernel	Filters
Max pool	1 × 2	/
Conv. 1	1 × 64	128
MsDCC1	1 × 3	32
MsDCC2	1 × 3	32
MsDCC3	1 × 3	64
TCN	1 × 3	64

Note: L = 100 samples; Δt = 0.01 s; K = 100 prediction steps; H = 1 s.

**Table 2:** Evaluation effects of different prediction methods.

Model	R^2^	MAE	MSE	MAPE (%)	RMSE
MsTCN	0.7349	1.5093	5.6262	67.3540	2.3719
LSTM	0.0015	2.7596	21.1929	78.6267	4.6036
TCN	−0.3217	3.4085	28.0538	75.8813	5.2966
TCN-LSTM	−0.4035	3.6105	29.7904	106.6727	5.4581
MsTCN-SE (proposed)	0.9915	0.2256	0.1804	11.4928	0.4247

**Table 3:** Evaluation effects of new indicators for different prediction methods.

Model	AR^2^	AMAE	AMSE	AMAPE	ARMSE
MsTCN	0.8674	0.6226	0.8124	0.4264	0.6046
LSTM	0.5007	0.3101	0.2935	0.3137	0.2327
TCN	0.3391	0.1478	0.0648	0.3411	0.1172
TCN-LSTM	0.2982	0.0973	0.0069	0.0332	0.0903
MsTCN-SE (proposed)	0.9958	0.9436	0.9941	0.9851	0.9292

**Table 4:** Comparison of real-time performance of each prediction model.

Prediction-Time Metric	MsTCN-SE	MsTCN	TCN	LSTM	TCN-LSTM
Single-prediction time (s)	0.410	0.292	0.061	0.059	0.230

## Data Availability

The data presented in this study are available on request from the corresponding author.
